# Determinants of patient choice of healthcare providers: a scoping review

**DOI:** 10.1186/1472-6963-12-272

**Published:** 2012-08-22

**Authors:** Aafke Victoor, Diana MJ Delnoij, Roland D Friele, Jany JDJM Rademakers

**Affiliations:** 1 NIVEL, Netherlands Institute for Health Services Research, P.O. Box 1568, 3500 BN, Utrecht, Netherlands; 2 Tilburg School of Social and Behavioural Sciences, Tilburg University, Tranzo, P.O. Box 90153, 5000 LE, Tilburg, Netherlands; 3 Centre for Consumer Experience in Health Care (CKZ), P.O. Box 1568, 3500 BN, Utrecht, Netherlands

**Keywords:** Choice behavior, Patient freedom of choice laws, Patient satisfaction, Healthcare providers, Quality indicators, Quality of healthcare, Healthcare reform, Review literature

## Abstract

**Background:**

In several northwest European countries, a demand-driven healthcare system has been implemented that stresses the importance of patient healthcare provider choice. In this study, we are conducting a scoping review aiming to map out what is known about the determinants of patient choice of a wide range of healthcare providers. As far as we know, not many studies are currently available that attempt to draw a general picture of how patients choose a healthcare provider and of the status of research on this subject. This study is therefore a valuable contribution to the growing amount of literature about patient choice.

**Methods:**

We carried out a specific type of literature review known as a scoping review. Scoping reviews try to examine the breadth of knowledge that is available about a particular topic and therefore do not make selections or apply quality constraints. Firstly, we defined our research questions and searched the literature in Embase, Medline and PubMed. Secondly, we selected the literature, and finally we analysed and summarized the information.

**Results:**

Our review shows that patients’ choices are determined by a complex interplay between patient and provider characteristics. A variety of patient characteristics determines whether patients make choices, are willing and able to choose, and how they choose. Patients take account of a variety of structural, process and outcome characteristics of providers, differing in the relative importance they attach to these characteristics.

**Conclusions:**

There is no such thing as the typical patient: different patients make different choices in different situations. Comparative information seems to have a relatively limited influence on the choices made by many patients and patients base their decisions on a variety of provider characteristics instead of solely on outcome characteristics. The assumptions made in health policy about patient choice may therefore be an oversimplification of reality. Several knowledge gaps were identified that need follow-up research.

## Background

In most European countries, patients were not encouraged to actively choose their healthcare provider. Patient choice has only recently gained importance in a number of northwest European countries, such as the Netherlands and the UK [1,2]. Important reasons for promoting patient choice were to reduce waiting times and to encourage competition between providers. Competition was expected to make care more responsive to patients and, among other things, improve efficiency (including cost decreases), quality and (in the UK) equity of healthcare [2-4]. In the Netherlands in 2006 for example, a demand-driven healthcare system was implemented to enhance competition between providers as a means of helping to achieve these goals. Another goal of emphasizing patient choice was to protect and promote the position of patients in healthcare [5]. It should be noted that some studies have shown that consumer-directed healthcare does not control costs better than other healthcare systems [6] and that its effects on quality are mixed [7]. This is, however, beyond the scope of this study.

The principle through which patient choice is assumed to bring about competition between healthcare providers is ‘voting with your feet’ [8]. This means that patients who are looking for high-quality care while minimizing costs will directly compare the prices and quality of different providers against each other and actively choose the provider that best fits their preferences and needs. In this context, ‘actively’ means that patients invest effort in acquiring information and making a conscious decision based on that information. If the money follows the patients, this selection process will encourage providers to compete for patients by improving their quality and decreasing their costs [9-12], which eventually helps ensure the quality, efficiency and equity of healthcare [11,13,14]. This line of reasoning applies not only to northwest European countries [2,3,5,15-17] but also to the USA, where patient choice was already an important element in the healthcare system [18].

For patients to be able to actively choose the best provider, they need to be informed about the quality of providers. Quality indicators were therefore developed. A quality indicator is a measurable aspect of care that gives an indication of the quality of care [19] and may concern the structure, process or outcomes of care delivered by a provider [20,21]. Structure indicators concern the organization of healthcare, whereas process indicators relate to the care delivery process and outcome indicators indicate the effect of the care delivered. Because patients have different information preferences, comparative information for all indicators is developed to enable patients to select the information that is relevant for them and to choose a provider based on that information [5,20].

Although patients are given a large amount of comparative information and are expected to choose the best provider based on this information plus information about prices, it is however questionable whether patients are indeed willing and capable to act as assumed. Questions arise such as whether patients do indeed actively choose their providers, whether they use the information provided, and whether a country’s health insurance system gives them enough opportunity and freedom to choose.

### Research focus

Although patient choice of healthcare providers is gaining importance in northwest European countries, it is not certain whether patients do behave as assumed. It is therefore high time that information is gathered on what is already known about this subject. In the current study, we are conducting a scoping review with the goals of describing the findings and range of research concerning patient choice of a wide range of healthcare providers in more detail (no studies were excluded based on the provider type) and of identifying knowledge gaps in the existing literature. We have not made selections or applied any quality constraints [22]. To our knowledge, not many studies exist that share this goal. This study is therefore contributing to the growing amount of literature on this subject. The three research questions we aim to answer are: (1) Do patients actively choose their healthcare providers? (2) How do patients choose their preferred healthcare provider? and (3) Which provider characteristics do they base their choice on?

## Methods

### Scoping review

We conducted a scoping review. A scoping review is a kind of literature review that is used when: a) a narrow review question cannot be defined; b) studies have employed a range of data collection and analysis techniques; c) no prior synthesis has been undertaken on the topic; and d) the reviewers are not going to assess the quality of the studies reviewed [23].

### Search strategy and selection of the literature

**Figure 1:**
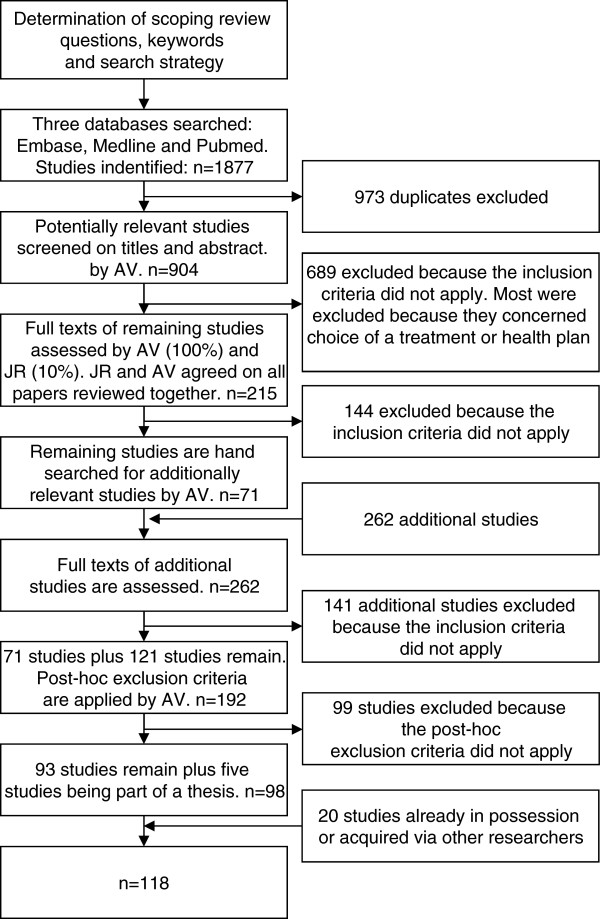
Search strategy and results.

The search was conducted on 17 August 2011 by one of the authors (AV). The databases used were Embase, Medline and PubMed. The keywords (i.e. patient, consumer, choice, provider, hospital, physician, doctor and their plurals) were determined after an initial broad search of the literature and consultations with a librarian and an expert on literature reviews. We decided to use a narrowly defined search string because otherwise the numerous irrelevant studies concerning choice of a health plan or treatment would outweigh the studies concerning patients’ choice of a provider. Only studies written in English were included, which can be justified by the observation that almost all references cited by the studies identified in the initial broad search were in English. This suggests that the most important sources are available in English. We only included studies from Western countries because the health insurance systems of other countries differ too much. For example, access to healthcare may be limited or healthcare services may not be well developed [24]. As healthcare systems have changed a great deal over past decades, we only included scientific papers from 1995 and later. The inclusion and exclusion criteria and the search string are shown in Table 1. This table also shows that post-hoc exclusion criteria were developed after a first review round and then applied in a second round. The development of such ‘post hoc’ criteria is central to the scoping review process as it is unlikely that researchers will be able to identify parameters for exclusion at the outset [23]. The selection method and search flow are represented in Figure 1.

**Table 1:** Inclusion and exclusion criteria and search string

	
Inclusion criteria	- written in English
	- concerns factors influencing patient choice or general choice theories regarding choices in health care
	- factors focused on are studied from a patient perspective or are determined by means of patient registration data analysis
	- does not solely concern the organization of a country’s health insurance system
	- reports empirical research (is not a commentary)
	- is a scientific paper
Post-hoc exclusion criteria	- reports
	- studies before 1995
	- studies from non-Western countries
Search string in PubMed	("patient choice"[TIAB] OR "patients choice"[TIAB] OR "patients' choice"[TIAB] OR "consumer choice"[TIAB] OR "consumers choice"[TIAB]) AND (provider[TIAB] OR providers[TIAB] OR hospital[TIAB] OR hospitals[TIAB] OR physician[TIAB] OR physicians[TIAB] OR doctor[TIAB] OR doctors[TIAB])

### Data extraction

A spreadsheet was created to chart the information that contributed to answering the research questions. Details of publication information, the choice situation, the study sample, the country in which the study took place and the kind of provider for which the preferences were assessed were recorded along with this information. This process was carried out by one of the authors (AV). The information extracted that helped answer the research questions was discussed with the other authors during team meetings in order to work towards an overall perspective on the factors emerging from the literature. Disagreements were discussed until a consensus was reached.

## Results

### Search flow

As shown in Figure 1, a total of 1877 publications were identified from the databases, of which 973 were duplicates. At the end of the selection process, 118 studies remained for further analysis (Figure 1). In Table 2, an overview of the characteristics of these studies is given.

**Table 2:** Characteristics of the included studies (n = 118)

**First author, year, country**	**Health care provider**^**1**^	**Respondents**^**2**^	**Primary method**	**Choice situation**^**3**^	**Type of provider characteristics influencing choice**^**4**^
Ahmad, 2002, Canada [110]	Family physician	O	Questionnaire	Hypothetical	S, P
Albada, 2009, Netherlands [36]	Hospital/ centre for ambulatory hospital care	P	DCE questionnaire	Hypothetical	S, P
Anell, 1997, Sweden [84]	Primary care physician, hospital & hospital specialist	O	Questionnaire	Hypothetical	S, P
Arora, 2004, USA [68]	GP	O	Experiment	Hypothetical	S
Bernard, 2006, USA [37]	GP	P	Questionnaire	Real	S, P
Boonen, 2009 ch.3, Netherlands [53]	Pharmacy	P	Patient registration data	Real	S
Boonen, 2009 ch.4, Netherlands [109]	Pharmacy	O	DCE questionnaire	Hypothetical	S
Boonen, 2009 ch.5, Netherlands [125]	GP	O	DCE questionnaire	Hypothetical	S
Boonen, 2009 ch.6, Netherlands [114]	GP/ Pharmacy	O	DCE questionnaire	Hypothetical	S
Bornstein, 2000, USA [75]	GP	P	Questionnaire	Hypothetical	S, P
Bouche, 2008, France [123]	Hospital	P	Patient registration data, questionnaire	Real	S
Bundorf, 2009, USA [103]	Fertility clinic	P	Patient registration data	Real	O
Burge, 2004, UK [82]	Hospital	P	DCE questionnaire	Hypothetical	S
Chalder, 2007, UK [138]	A&E department	P	Patient registration data, questionnaire	Real	P
Chandler, 2000, USA [25]	Obstetrician-Gynaecologist	P	Questionnaire	Hypothetical	S, P
Cheraghi-Sohi, 2008, UK [26]	GP	P	DCE questionnaire	Hypothetical	S, P
Chernew, 1998, USA [27]	Hospital	P	Patient registration data	Real	S, O
Combier, 2004, France [28]	Maternity hospital	P	Interview	Real	S
Cooper, 1996, USA [69]	Individual Physician	P	Interview, patient registration data	Real	S
Cutler, 2004, USA [29]	Hospital	P	Patient registration data	Real	O
Damman, 2009, Netherlands [31]	Hospital	O	Semi-structured cognitive interviews	Hypothetical	O
Damman, 2010, Netherlands [106]	NA	O	DCE questionnaire	Hypothetical	NA
Damman, 2011, Netherlands [119]	Hospital	O	DCE questionnaire	Hypothetical	S, P
Dawson, 2004, UK [56]	Hospital	P	Patient registration data	Real	P
Dawson, 2007 UK [57]	Hospital	P	Patient registration data	Real	P
De Boer, 2011, Netherlands [129]	General	P	Questionnaire	Hypothetical	P
De Groot, 2011, Netherlands [41]	Hospital	P	DCE questionnaire	Combination	S, P
Dealy, 2005, UK [117]	Hospital	NA	Literature review	NA (review)	S
Dijs-Elsinga, 2010, Netherlands [30]	Hospital	P	Questionnaire	Combination	S, P
Dixon, 2010, UK, Netherlands [2]	Hospital	NA	Analysis of secondary literature and primary data	NA	S, P, O
Exworthy, 2006, UK [59]	General	NA	Literature review	NA (review)	S, P, O
Faber, 2009, Netherlands [60]	General	NA	Literature review	NA (review)	*
Fasolo, 2010, UK [92]	Hospital	O	Focus group, questionnaire	Hypothetical	S, P, O
Finlayson, 1999, USA [51]	Hospital	P	DCE interview	Hypothetical	S, O
Foster, 2010, Australia [91]	NA	NA	Literature review	NA	NA
Fotaki, 2008, UK [16]	Hospital & GP	NA	Literature review	NA (review)	S, P
Fung, 2005, USA [18]	Primary care physician	P	DCE questionnaire	Hypothetical	P, O
Fung, 2008, USA [105]	General	P	Literature review	Real	O
Geraedts, 2007, Germany [74]	Hospital	P	Interview	Hypothetical	S, P, O
Gooding, 1995, USA [107]	Hospital	O	Questionnaire	Hypothetical	NA
Groenewoud, 2008 ch.2, Netherlands [99]	General	O	Literature review, interviews, document analysis	NA (review)	S, P
Groenewoud, 2008 ch.3, Netherlands [54]	General	NA	Literature review	NA (review)	S
Groenewoud, 2008 ch.4, Netherlands [98]	General	P	Grounded theory approach	Real	S, P, O
Groenewoud, 2008 ch.5, Netherlands [32]	General	P	Q-methodology, questionnaire	Hypothetical	S, P, O
Groenewoud, 2008 ch.6, Netherlands [113]	General	P	DCE questionnaire	Hypothetical	S, P, O
Groenewoud, 2008 ch.7, Netherlands [61]	General	P, O	Concept mapping	Hypothetical	S, P, O
Grytten, 2009, Norway [3]	GP	P	Interview or questionnaire, patient registration data	Real	S
Guile, 2007, USA [52]	Obstetrician-Gynaecologist	P	Interview	Hypothetical	S, P
Harris, 2003, USA [42]	Individual physician	P	Questionnaire	Combination	NA
Haynes, 2003, UK [38]	GP practice	P	Patient registration data	Real	S
Hibbard, 1997, USA [89]	Health plan report cards	NA	Literature review	NA (review)	NA
Hibbard, 2003, USA [93]	General	NA	Literature review	NA (review)	NA
Hibbard, 2010, USA [100]	General	O	Questionnaire	Hypothetical	NA
Hirth, 2000, USA [139]	Nursing home	P	Patient registration data	Real	NA
Hirth, 2003, USA [65]	Nursing home	P	Patient registration data	Real	S, O
Hodgkin, 1996 USA [140]	Hospital	P	Patient registration data	Real	S
Hoerger, 1995, USA [86]	Prenatal care physician	P	Interview, patient registration data	Real	S
Howell, 2002, USA [77]	Obstetrician	P	Interview, patient registration data	Real	S, P
Humphreys, 1997, Australia [127]	GP	O	DCE questionnaire or interview, patient registration data	Hypothetical	S, P
Johnson, 2005, USA [43]	Obstetrician-Gynaecologist	P	Questionnaire	Combination	S, P
Kerssens, 1997, Netherlands [55]	Thirteen different health professions (individuals).	O	Questionnaire	Hypothetical	S, P
Ketelaar, 2011, Netherlands [141]	Hospital	NA	Literature review	NA (review)	*
Kiiskinen, 2010, Finland [83]	Dentist	O	DCE questionnaire	Hypothetical	S, P
Kolstad, 2009, USA [62]	General	NA	Literature review	NA (review)	S, P, O
Kooreman, 2010, Netherlands [88]	NA	NA	Literature review	Hypothetical	NA
Laamanen, 2010, Finland [44]	Individual doctor	P, O	Questionnaire	Combination	S
Lako, 2009, Netherlands [79]	Hospital	P	Questionnaire	Real	S, P
Lambrew, 2005, USA [85]	General	O	Interview	Hypothetical	NA
Lubalin, 1999, USA [63]	General	NA	Literature review	NA (review)	NA
Lux, 2011, Germany [45]	Hospital	P	Questionnaire	Combination	S, P, O
Magee, 2003, UK [64]	General	P, O	Focus group	Hypothetical	S, P, O
Marang-van de Mheen, 2010, Netherlands [46]	Hospital	P	Questionnaire	Combination	S, P
Marang-van de Mheen, 2010, Netherlands [33]	Hospital	P	DCE questionnaire	Hypothetical	S, P, O
Mavis, 2005, USA [126]	GP, ob-gyn & surgeon	P	Questionnaire	Hypothetical	S, P
McGlone, 2002, USA [76]	GP	P	Questionnaire	Real	S, P
Merle, 2009, France [67]	Hospital	P, O	Questionnaire or interview	Hypothetical	S, O
Moodie, 2008, UK [142]	Surgeon performing a cataract surgery.	P	Questionnaire	Hypothetical	S, P
Morrison, 2003, Australia [34]	GP	O	Questionnaire	Hypothetical	S, P
Moser, 2010, Netherlands [90]	Hospital	P	Cognitive interview, focus group	Hypothetical	NA
Mukamel, 1998, USA [104]	Hospital & Surgeon	P	Patient registration data	Real	O
Mukamel, 2001, USA [102]	NA (review)	NA	Literature review	Real	S
Newton, 2007, Australia [115]	Medical facility/ GP	O	Questionnaire	Hypothetical	S, P
Nguyen, 2006, Finland [39]	Dentist	P	Questionnaire, patient registration data	Real	S
Orr, 1998, UK [66]	Excimer laser treatment centre	P	Questionnaire	Real	S, P
Peters, 2007, USA [96]	Hospital	O	DCE questionnaire	Hypothetical	NA
Peters, 2009, USA [95]	Hospital/ health plan	O	DCE questionnaire	Hypothetical	NA
Petry, 2004, USA [143]	Health Care Practitioner (institution and individual)	P	Questionnaire	Real	S
Plunkett, 2002, USA [70]	Obstetrician/ Gynaecologist	P	Interview	Real	S, P
Propper, 2007, UK [144]	Hospital	P	Patient registration data	Real	S
Rademakers, 2011, Netherlands [80]	General	P	Secondary analysis on questionnaire and interview data	Real	S, P
Redelmeier, 1995, USA [94]	NA	O	DCE questionnaire	Hypothetical	NA
Reyna, 2009, USA [97]	NA	NA	Literature review	NA (review)	NA
Ringard, 2011, Norway [130]	Hospital	P	Patient registration data, questionnaire	Real	P
Robertson, 2008, UK [128]	GP	P	Questionnaire	Real	S, P
Robertson, 2011, UK [47]	Hospital	P	DCE questionnaire	Combination	S, O
Roh, 2005, USA [121]	Hospital	P		Real	S
Roh, 2008, USA [120]	Hospital	P	Patient registration data	Real	S
Rosenthal, 2009, USA [73]	Individual physician.	P	Patient registration data	Real	S
Ryan, 2000, UK [35]	Hospital	O	DCE questionnaire	Hypothetical	S, P
Safran, 2001, USA [116]	Individual physician.	P	Longitudinal: questionnaire, patient registration data	Real	S, P
Saha, 2000, USA [145]	General	P	Interview	Real	S
Scanlon, 2008, USA [40]	Hospital	P	Patient registration data, questionnaire	Real	S, O
Schauffler, 2001, USA [101]	Hospital	NA	Literature review	NA (review)	O
Schnatz, 2007, USA [78]	Obstetrician/ Gynaecologist	P	Interview	Hypothetical	S, P, O
Schneider, 1998, USA[48]	Hospital	P	Interview	Combination	S
Schwartz, 1999, USA [134]	NA	O	DCE questionnaire	Hypothetical	NA
Schwartz, 2005, USA [49]	Hospital	P	Interview	Combination	S, O
Shah, 2010, UK [112]	Hospital	P	Questionnaire	Hypothetical	S, P
Siciliani, 2007, UK [58]	Hospital	P	Patient registration data	Real	P
Sinaiko, 2011, USA [108]	Physician	O	DCE questionnaire	Hypothetical	O
Tai, 2004, USA [111]	Hospital	P	Patient registration data, questionnaire	Real	S
Van Empel, 2011, Netherlands, Belgium [50]	Fertility clinic	P, O	DCE questionnaire	Combination	S, P, O
Varadarajulu, 2002, USA [71]	Endoscopist	P	Questionnaire	Hypothetical	S, P
Varkevisser, 2007, Netherlands [118]	Hospital	P	Patient registration data	Real	S, P
Varkevisser, 2009, Netherlands [81]	Hospital	P	Patient registration data	Real	S, O
Varkevisser, 2010, Netherlands [122]	Hospital	P	Patient registration data	Real	S, P
Vonberg, 2008, Germany [124]	Hospital	O	Interview	Hypothetical	S, P, O
Zuckerman, 2002, USA [72]	Obstetrician/ Gynaecologist	O	Questionnaire	Hypothetical	S

^1^*NA* = not applicable; ^2^*P* = patients; *O* = other; *NA* = not applicable; ^3^*Real* = patients in a real choice situation; *Hypothetical* = no real choice situation; *Combination* = both a real and a hypothetical choice situation; *NA* = not applicable; ^4^ *S* = structure indicator(s); *P* = process indicator(s); *O* = outcome indicator(s); *NA* = not applicable; * = no provider characteristics found that influence choices.

### Study characteristics

#### Study sample and choice situation

Most studies (n = 70) used only patients as participants, e.g. [25-30]. Other studies looked at the general (adult) population, or a specific subclass of the population such as those in work or with insurance, the elderly or people of a specific ethnicity or gender.

For the choice situation, the majority of studies (n = 49), e.g. [31-36], used discrete choice experiments or questionnaires asking participants about potential choices and preferences, while somewhat fewer studies investigated patient choice in real choice situations (n = 43), e.g. [27,28,37-40]. Only a few studies combined the analysis of real choice situations with experiments or questionnaires (n = 11) [30,41-50].

#### Countries

The majority of studies into patient choice took place in the USA (n = 51), e.g. [18,25,27,29,51,52], followed by the Netherlands (n = 29), e.g. [30,31,46,53-55], and the UK (n = 19), e.g. [26,35,38,56-58]. Countries with less research on the subject are Canada, France, Australia, Finland, Sweden, Norway, Belgium and Germany. There are two areas that studies from the USA examined relatively more often than those from Europe: revealed preference research (based on analysis of registration data) about the use of comparative information, and research into the influence of health plans on patients’ choices.

#### Kind of provider

Many studies do not focus on a particular kind of healthcare provider, but focus on several types of healthcare provider or do not specify what they are focusing on (n = 25), e.g. [59-64]. Of the studies that do focus on a particular kind of provider, choice of healthcare institutions (n = 54), e.g. [27,29,31,65-67], has been investigated more often than choice of individual providers (n = 31), e.g. [68-73]. Most studies that investigated the choice of an institution were investigating the choice of a hospital (n = 46), e.g. [27,29,31,51,57,74]. Of the studies investigating the choice of an individual provider, most concerned the choice of a GP, family physician or primary care doctor (n = 12), e.g. [3,18,37,68,75,76], followed by the choice of an obstetrician or gynaecologist (n = 7), e.g. [43,52,70,72,77,78].

### First research question: do patients actively choose their healthcare providers?

Research shows that few patients actively choose their healthcare provider [16,30,41,47-49,64]. For example, Schwartz (2005) found that only ten per cent of patients seriously considered an alternative to their local hospital when undergoing surgery [49]. Generally, patients rely on their GP to choose for them [2,41,49,67,70,79,80] or go to the nearest provider [27,59,81]. Furthermore, patients rely on their previous healthcare experiences when deciding where to receive care [25,46,47,49]. This seems to apply to both Europe and the USA (for those patients who can choose). However, certain patient groups (such as more highly educated and younger patients [59,79,80,82,83], patients with higher incomes [59,82,83] and patients without an existing (satisfactory) relationship with a provider [42,47]) make an active choice more often.

According to several studies, a substantial fraction of the patients does not consider choice to be very important [16,43,64,84,85]. Consequently, these patients are less likely to make an active choice. Even so, they find choosing a GP or hospital more important than choosing a hospital specialist [84]. The importance patients attach to choice differs between patient groups. For example, according to one study, older patients, female patients, those who live further away from a hospital, less highly educated patients and those with a bad experience with their local hospital are more favourably inclined towards the free choice of hospital [47]. A second reason for patients not to choose actively is that the degree of choice they experience or their ability to exercise their choice is limited. For example, patients’ perceived degree of choice or ability to choose was found to be influenced positively by family income [16,85,86], general state of health [85] and willingness and ability to travel [16], and negatively by restrictions imposed by health insurers [85,86], age and female gender [16]. Additionally, some studies found that some patient groups are more likely to be offered a choice of provider by their GP than other patient groups, e.g. Caucasians [2], healthier patients and patients who need an operation or hospital admission [47].

### Second research question: how do patients choose their preferred healthcare provider?

#### Patients’ decision-making processes

Policy makers assume that patients selectively choose high-quality providers based on weighing up the information about the different providers: in other words, that they make a rational choice [87]. For patients to be able to choose as this assumes, they need complete information, unrestricted cognitive abilities, consistent preferences, willpower and the ability to foresee their needs [88]. However, several studies suggest that these conditions are rarely satisfied [88-90] and most patients are consequently unable to make a completely rational choice [38,63,88,91-93]. This results in choices based on only some of the provider characteristics and/or irrelevant factors such as their current mood [31,63,88-91] and often to no choice at all [88,93,94]. According to several studies, the degree to which patients are capable of processing the information rationally is influenced by their health literacy (the degree to which they have the capacity to obtain, process and understand the basic health information needed to make appropriate health decisions) and their numeracy (the ability to apply numbers as needed to manage your health) [60,92,95-97]. For example, low numeracy leads to people being influenced more often by factors that are irrelevant to the choice problem.

Furthermore, a patient’s activation level (i.e. the extent to which patients seek and use healthcare information and actively choose between providers) also influences patients’ choice processes, according to several studies. Some patients actively search for providers, while others rely on their GP for advice [42,62,64,76,86,98]. How active patients are depends on their characteristics [42,47,76,86,98]. For example, patients who do not have a strong tie or have an unsatisfactory tie to individual physicians [42,47] are more active consumers. Patients who make more active choices may make use of systematic reasoning using all available information or may make a more intuitive choice using only subsets of the information [31,90,92]. Low numeracy leads to less use of systematic reasoning [92]. However, only a few patients systematically process all information, according to Damman [31].

#### Use of information sources

Research shows that patients use various information sources in their decision-making processes. Comparative information is one example of an information source. Findings on whether patients see the relevance of comparative information are mixed (i.e. mutual inconsistency between the studies). One reason for patients finding this information irrelevant is that they expect a high standard everywhere and are unwilling to ‘shop around’ [16,49]. Often, patients who do find this information relevant eventually do not use it, which suggests that there is a difference between what patients say and what they actually do [16,31,64]. This difference is confirmed by research that directly compared revealed preferences against stated preferences [30,45,46,48,49]. Patients use more comparative information in future choices and in advice to others than they used in previous choices. Reasons for not using it are that they encounter barriers to its use, e.g. the short time frame in which to select a provider and geographical barriers [62], unavailability of the right information [31,74,76,84,90,99], distrust of the information [49], information overload [31,60,100] and an insufficiently clear presentation of the information [30,31,60,92,100,101]. So, although patients indicate that they find comparative information important, research suggests that relatively few patients make use of comparative information, are aware of its existence or understand it [16,31,48,62,64,102]. This applies in both Europe and the USA. Patients appear to use comparative information only in certain circumstances, such as when there is a single outcome of major importance and the data can be easily understood, or in the absence of a meaningful and trusting doctor-patient relationship [16,60]. Patients with low health literacy in particular find insufficiently clear presentation formats more of a problem [60,95,96]. Nevertheless, according to a few revealed preference studies from the USA, the release of comparative information does result in small changes in providers’ market shares [29,62,103-105]. However, this effect may be caused by factors other than patients who are actively choosing, for example GP referrals. Finally, research indicates that explicitly giving or making patients aware of comparative information [52,62,78] and improving the presentation format [63,92,95,97,100,106] increases its use.

Research shows that patients use other information sources more often than comparative information. A patient’s own previous care experience, for example, is the most important information source for many patients [42,45,62,107,108]. A positive experience with a particular provider positively influences the future choice for that provider [25,30,44,45,47,109]. Patients’ general care experiences also influence their choices. For example, two studies found that positive experience with female physicians positively influences patient preference for a female physician [72,110] and that patients who had bypassed their closest rural hospital once are more likely to bypass it again [111]. Social influence (e.g. a provider’s general reputation, the influence of someone’s referring physician or the recommendations of friends and acquaintances) is a third important information source [46,59,66,67,76,112]. However, different studies find different effects of this information source. Only the influence of a referring physician has a consistent strong positive effect.

Which of these information sources are used differs between patients [28,42,45,86,108,113]. For example, older [28,42] and less highly educated patients [113] are more likely to follow the advice of their physician. Older, less highly educated, less literate [60,84,92,106] patients and those already in the healthcare system [62] generally use less comparative information.

### Third research question: which provider characteristics do patients base their choice of healthcare provider on?

Because the nature of this research question is suitable for quantitative analysis, we quantitatively analysed the studies that investigated the influence of provider characteristics on patients’ choices. In 101 studies, the influence of provider characteristics on patients’ choices was investigated. The structure-process-outcome model of quality care [21] is used in this review in order to summarize the characteristics influencing this theme. The factors studied most often are those related to structure (n = 86), followed by process (n = 60) and outcome (n = 43). Because of the relatively large amount of literature on structure, we have paid more attention to this factor. The importance that patients attach to the different factors differs between patients, depending on their socio-demographic (n = 44) and disease (n = 31) characteristics and their knowledge, attitudes and beliefs (n = 12). When we discuss the specific provider characteristics below, we will only go into detail about the influences that have been investigated relatively often. Given the large number of sources included in this review, for the sake of manageability we will cite no more than six at a time.

#### Structure

Seven factors can be distinguished for the structure aspect, namely the availability of providers, the accessibility of the providers, the type and size of the providers, the availability/experience/quality of the staff, the organization of healthcare, the cost of treatment and socio-demographic factors of the individual doctors.

Availability (n = 29): it was commonly reported that the availability of providers influences choice (n = 18). Some patients have only a few providers to choose from and for some patients the number of providers they can actually choose from is limited because of, for example, language difficulties [2,3,16,48,65,102]. Whether or not a given provider is available for patients depends on their insurance plan, especially for patients in the USA. If patients have to make co-payments or do without certain benefits when receiving care from a particular provider, they are less likely to choose that provider (n = 10) [40,53,69,73,86,108]. This incentivizing by insurers does not affect all patients’ decisions equally. Examples of observed effects are that being female [53] or having a lower income [73,109] positively affect, and that already having a provider [114] or being in poor health [73] negatively affect responsiveness to insurer incentivizing.

Accessibility (n = 55): the issue most discussed is distance or convenient location (n = 50). Generally, patients are averse to travel time and prefer a provider that is close by and not abroad (n = 44) [30,66,67,82,111,115]. Another important issue is that patients prefer a provider that is accessible by their own transport or public transport (n = 11) [28,30,38,64,112,116]. Other issues are parking (n = 4) [2,30,46,112] and transport that is organized or paid for (n = 4) [16,59,82,117]. Studies found a positive relationship between age and the importance of distance, easy access by transport and parking facilities (n = 12) [30,38,51,82,111,118]. Furthermore, being more highly educated (n = 8) [30,47,51,82,111,119] and being willing to travel (n = 3) [47,59,64] negatively influence the importance attached to distance. The specific disease influences the importance attached to distance (n = 6) [30,59,81,119-121], e.g. distance is more important for patients who need cataract surgery than for patients who need hip or knee surgery [119].

Type and size of the institution (n = 37): the issue most discussed was provider ownership/affiliation (n = 17). It was generally found that this aspect influences choice (n = 15) [44,65,74,120-122]. For example, research indicates that patients prefer an individual provider that is affiliated to an (academic) hospital [62,70]. Besides, American patients prefer private, non-profit providers over public and commercial ones [27,65,120,121], whereas patients from the UK prefer public hospitals [66]. However, findings are mixed on whether patients prefer a university medical centre [45,81,118,122]. Two studies found that patients prefer a university medical hospital [45,81], while two others found that they do not [118,122]. Two other important issues are the range and quality of facilities (n = 22) [30,61,74,111,120,121] and the provider size (n = 11) [27,30,75,111,121,122]. Patients generally prefer clean hospitals with complex, high-quality services. Findings on preferred provider size are mixed. For example, Bouche found that patients were more likely to choose low-volume hospitals [123], while the number of beds does not influence choice of hospital according to Roh [120]. Bornstein found that patients prefer GP practices with several doctors [75]. Comparison of the studies reviewed could not let us show why findings are mixed, as there are so many differences between them. Examples of differences are the kind of healthcare provider that studies focused on and the methods used to acquire patients’ preferences.

Staff (n = 35): a large number of studies found that the medical qualification/expertise of providers is an important determinant of choice (n = 27) [52,77,78,86,109,112]. Patients prefer providers with a quality certificate and qualified physicians. Furthermore, patients prefer experienced providers (n = 10) [30,33,43,52,70,113]. Yet other factors that patients prefer are that the provider’s specialization/interest fits their care needs (n = 6) [37,59,64,70,75,119] and the availability of sufficient staff per patient (n = 3) [62,113,124].

Organization of healthcare (n = 27): some of the factors that positively influence the preference for a provider are related to the organization of healthcare [45,53,59,61,75,98]:

1) whether you can be treated at a convenient time or place or by the doctor of choice (n = 15) [36,53,75,86,119];

2) actions to improve service quality and efficiency (n = 12) [76,83,113,115,125,126]. Aspects in this category are regularly inviting patients for checkups, making house calls, providing bulk billing services, having practice assistants available, spending enough time on personal care, and complaint handling;

3) whether a provider is accessible by phone and Internet (n = 5) [66,86,109,127,128].

Costs (n = 12): the evidence about the influence of cost on choice is mixed [26,28,69,75,86,113]. Differences may be caused by whether the care provided by a certain provider is insured or not, as the cost of treatment generally only influences choice when patients also have to make payments themselves. For example, Combier (2004) found that women do not take costs into account when choosing a maternity hospital because they do not have any out-of-pocket expenses [28], whereas research by Kiiskinen (2010) indicates that patients do take out-of-pocket costs into account when choosing a dentist [83].

Socio-demographic factors (n = 18): the two most extensively studied factors are gender (mostly whether the direct care provider has the same gender as the patient) (n = 16) and age (n = 7) of the provider [37,43,52,75,76,84]. It is generally found that a physician’s demographic parameters do influence choice, but that other factors are usually perceived to be more important [25,37,43,70,76]. This is confirmed by the finding that explicitly giving or making patients aware of comparative information reduces the influence that variables such as the age and gender of the individual providers have on choice [52,62,78]. The characteristics that patients attribute to women, such as positive social skills, positively influence their preferences for women [25,55,110].

#### Process

Five factors can be distinguished for the process aspect, namely interpersonal factors, availability of information, continuity of treatment, waiting time and the quality of treatment.

Interpersonal factors (n = 40): the issue most discussed was the physician’s communication style (n = 36). Most studies found that this factor influences choice (n = 36) [45,62,66,78,92,115]. Generally, patients prefer a provider with a friendly and understanding communication style who listens to the patient and with whom the patient has a good relationship or feels a personal click. Other factors that are found to influence choice positively are whether the patient is involved in decision making about care (n = 12) [26,34,37,62,76,99] and a friendly provider atmosphere (n = 7) [30,32,33,46,62,76]. Age positively influences the importance attached to interpersonal characteristics according to several studies (n = 6) [26,30,34,76,119,126], while education negatively influences the importance of interpersonal characteristics (n = 6) [26,30,33,34,76,126]. Research into the influence of disease characteristics shows that patients with more complex or severe diseases attach more importance to interpersonal characteristics [26,50,113,129] and that the specific disease influences the importance the patient attaches to interpersonal characteristics [30,80,98,129].

Information provision (n = 10): most studies found that whether and how information is provided is a determinant of choice (n = 7) [30,36,59,61,99,119]. Continuously giving relevant information during and before treatment has a positive influence on choice.

Continuity (n = 10): being able to keep seeing the same doctor has a positive influence on the choice of provider [26,34,36,99,116,127].

Waiting time (n = 30): most studies found a negative influence of the time spent on waiting lists and time in the waiting room (n = 27) [26,30,35,46,59,130]. However, the specific disease influences the importance a patient attaches to waiting time (n = 4) [30,33,80,119].

Quality of treatment (n = 12): this factor has to do with the quality of the medical treatment (n = 8). All studies found at least some positive influence of this factor on choice [26,30,41,61,99,119]. Examples are whether medical treatment is high quality and whether care is delivered as agreed, the number of cancelled operations and whether patients have a clear care plan. Additionally, three studies show that the rules or activities implemented in order to deliver good care are an important issue, e.g. the clinical standards used, whether care is interdisciplinary, and the protocols and procedures a provider has implemented [45,61,66].

#### Outcome

Although many studies (n = 30) found that outcome indicators such as mortality or pressure sore rates had a strong or moderate influence on choice [18,27,50,64,98,102], about half that number (n = 15) found that the influence was weak or that there was no influence at all [16,46,48,54,64,102]. Generally, other characteristics are found to be more important than outcome, such as GP referral and distance [16,30,41,46,64,67]. Differences in the importance attached to outcome indicators are partly explained by the differences between the characteristics that patients say are important and the ones they act upon in a real choice situation. These differences have often been uncovered by research that directly compared revealed preferences against stated preferences [62]. For example, patients indicate that they are willing to use more quality information items, including outcome indicators, in future choices than they actually used in previous choices [30,46,48,49]. Additionally, outcome indicators influence the advice they would give to friends, whereas they did not have a strong influence on their own previous choices [45,49]. It is however difficult to indicate whether this phenomenon accounts for all the inconsistencies in the findings between the studies reviewed, as there are also many other differences between them. Several studies (n = 10) found a positive relationship between the level of education and the importance attached to outcome characteristics [28,33,67,113,119,124]. Patients with more complex or severe diseases attach less importance to outcome characteristics (n = 2) [29,113] and the specific disease influences the importance that the patient attaches to outcome characteristics (n = 7) [30,33,45,46,98,119].

## Discussion

Choice of a healthcare provider does not seem to be as straightforward a process as is sometimes assumed in health policy, i.e. that patients look for high-quality care while minimizing cost and ‘vote with their feet’ by choosing the provider that best fits their needs and preferences [2,11,13,18,131,132]. As this review shows, whether and how patients choose a provider and their eventual choices are determined by the interplay between patient and provider characteristics. This review has answered three questions.

The first research question concerns whether patients actively choose their healthcare providers. Research indicates that patients do not generally choose actively [47,49]. Reasons are that a substantial proportion of patients do not find choice very important [16,64,84,85], that the degree of choice for some patients is limited [2,16,47,85,86] and that the available information is not enough or unsuitable to base decisions on [30,31,60,92,100,101]. Especially because of the last two factors mentioned, there is a difference between the characteristics that patients state as being important and the characteristics they act upon in a real choice situation. The second research question is about how patients choose. Policy makers assume that patients, as they aim for high-quality care while minimizing costs, will actively choose the best provider. However, research shows that most patients are unable and/or unwilling to make a completely rational choice. This is supported both by research in healthcare (e.g. health plans, treatments, and health-related behaviour) and in other areas (e.g. personal finance, which school to attend) [133-137]. Instead, choices are based on only some of the provider characteristics [31,63,88-91] and patients choose a provider that is good enough, or make no active choice at all [88,93,94]. Furthermore, their degree of activation [42,62,64,76,86,98], the information sources they use and how systematically they compare the information about the characteristics of the various providers also differ [31]. Apparently, most patients do not look for the highest quality, as only a few go systematically through all the comparative information [31]. Instead, they only take information into account that confirms their expectations, they often stay with their current provider [25,90] and they rely on others’ experiences [108] or their GP’s advice [98,117]. Finally, in the investigations for the third research question, namely the provider characteristics that patients base their choices on, it transpires that patients base their choices on a variety of structural, process and outcome quality indicators. In fact, structure and – in particular – process indicators are more important than outcome indicators [50,80]. The importance attached to the different characteristics differs between the various patient groups.

Because the USA has a longer history than countries in Europe [64] of competition in various areas and of publishing information on the quality of care among different providers, it might be expected that American patients would make more active choices for high-quality providers. However, in practice, the choices made by both European and American patients are determined by a complex interplay between a variety of patient and provider characteristics and different patients make different choices - generally passive ones - in different situations. Nevertheless, differences between the choice processes and choices of American and European patients do exist, often resulting from the distinct healthcare systems of the two continents. For example, in the USA, insurers traditionally have an important role as prudent buyers of care on behalf of their members and research suggests that they partly determine the specific providers that are available to patients [86].

### Differences between studies

Scoping reviews analyse studies that use a range of data collection techniques. Different techniques may lead to different results. For example, it is to be expected that results from stated preference research differ from those from revealed preference studies. For outcome indicators, for example, most studies investigating hypothetical choices found that outcome indicators influence patients’ choices. However, most studies investigating real choices found that outcome indicators have a limited influence on patients’ choices. This difference is confirmed by research that directly compared revealed preferences against stated preferences [30,45,46,48,49]. Exceptions are results from studies analysing patient registration data. Most studies found that more patients are admitted to providers that perform better (on outcome indicators) and fewer to providers performing less well. However, this effect may be caused by factors other than patients choosing actively, for example by GP referrals.

It is also to be expected that the characteristics patients consider to be important will differ for individual providers and institutions. Fung (2008), for example, found that public reporting of performance data did not affect selection of hospitals, while it did affect selection of individual providers [105]. Interpersonal indicators are also found to influence choice of an individual provider more often than choice of an institution. These differences can, however, partly be explained by the research methods used in the specific studies. Studies investigating the choice of individual providers study the importance of interpersonal indicators more often. For example, Newton (2007) found that patients focus on interpersonal factors when choosing a GP but not when choosing a medical clinic facility. Patients’ perceived importance of interpersonal indicators was, however, not investigated when choosing a medical clinic facility [115]. This underlines the difficulty of indicating the exact causes of the differences found between the studies under review, as there are numerous differences in their data collection and analysis techniques.

### Knowledge gaps

We identified several knowledge gaps. Firstly, despite the fact that there is an increasing amount of literature from behavioural economics and psychology, the behavioural economics of provider choice have received relatively little attention compared to the literature, which assumes that patients choose their providers more or less rationally. Although policy makers assume that patients’ information processing proceeds rationally, the results of several studies suggest that patients are often not capable of making rational choices [136]. This also indicates the relevance of the context in which the relationships occur that were found by the studies. Many studies do not explicitly address the issue that their findings may depend on the specific decision-making context, e.g. that they focus on a hospital or GP, that they asked for patients’ preferences or the attributes they based their decision on, whether patients were ill or not, etcetera. We recommend that researchers should specify the influence of the research context on the research findings and explain any discrepancies between their findings and the findings of other studies, given the differences in context. A final gap in the current state of knowledge is that relatively few studies analysed choice in a real choice situation, instead using an experimental design. More research should be conducted into the provider characteristics that patients take into account in real choice situations, especially because preferences are not static but depend on the decision context. As this review shows, there is a difference between the factors that patients say they find important and the ones they actually base their decisions on. However, we are aware of the difficulty of setting up such a study.

### Strengths, limitations and follow-up research

A strong point of this review is that it has a broad scope and attempts to draw a picture of how patients choose healthcare providers and what determines their choice. We have tried to point out the factors that are important determinants of patient choice according to the existing literature, without making selections or excluding any studies because of their lower quality. Additionally, the search and inclusion process, which included developing a search strategy in consultation with a librarian and literature review expert and having two reviewers for a proportion of the entire source texts, is a strong point.

One limitation of this review is that its scope may not be broad enough because only scientific papers were included. Additionally, because of our narrow search string, we may have missed some relevant papers on the subject. However, the papers that we read in a later stage of the review did not add any significant new insights. Furthermore, the range of data collection and analysis techniques used in the studies under review makes them hard to compare and makes the mixed results hard to interpret. The results of any particular reviewed study may have been influenced by the exact kind of provider and provider characteristic studied and the method used for obtaining the data. For example, Groenewoud (2008) found that GP recommendations do not influence choices much, whereas Plunkett (2002) found that they do. The latter analysed real choice situations and the former asked for patients’ preferences regarding certain provider characteristics [32,70]. However, other aspects also differed between the two studies, so we could not clarify this mixed result.

A related issue is that a scoping review cannot present absolute truths, because no exhaustive search has been done and we did not conduct a quality assessment of reviewed sources. The results should therefore be interpreted with some caution. Nevertheless, due to the large number of studies included, we believe that the current review provides a thorough survey of the available literature on the factors that influence patient choice and the range of research conducted into the subject.

## Conclusion

Patients’ choices are determined by a complex interplay between a variety of patient and provider characteristics. There is no such thing as the typical patient: different patients make different choices in different situations. Patients often attach greater importance to their own previous healthcare experiences or to GP recommendations than to comparative information. Additionally, patients base their decisions not only on outcome indicators but on a variety of provider characteristics. It can thus be argued that the choice process is much more complex than is often assumed. This is true for both Europe and the USA. Most patients are unable and/or unwilling to make a completely rational choice [134-137]. A number of gaps in current knowledge were identified.

## Competing interests

The authors declare that they have no competing interests.

## Authors’ contributions

AV participated in the design of the study, carried out the literature search and selection process, charted and modelled the data and drafted the paper. JR also participated in the design of the study, the literature selection process and the modelling of the data and helped to draft the paper. All the authors participated in modelling the data, drafting the paper and reading and approving the final text.

## Pre-publication history

The pre-publication history for this paper can be accessed here:

http://www.biomedcentral.com/1472-6963/12/272/prepub
